# Supplementary material for: Oral Chagas disease outbreak by bacaba juice ingestion: A century after Carlos Chagas’ discovery, the disease is still hard to manage
Source: PLoS Negl Trop Dis. 2024 Sep 18;18(9):e0012225. doi: 10.1371/journal.pntd.0012225 (PMC11441692; doi:10.1371/journal.pntd.0012225)
Supplement: S1 Fig — (DOCX) [file pntd.0012225.s001.docx]

**S1 Fig***.* Electrophoretic profiles of PCR products of the variable region of the kDNA minicircle obtained with DNA from some clinical samples of patients with Chagas disease.


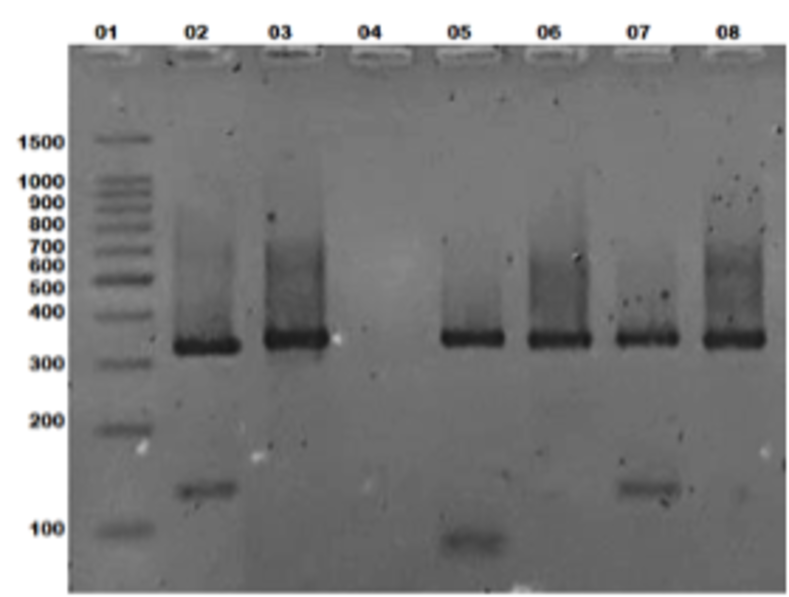


**Captions**: **Line 1:**100 bp DNA ladder molecular size marker (Promega, USA). Profiles showing a positive amplification reaction for the fragment of 330bp of the variable region of the kDNA minicircle. Line 2, profile of positive control sample for Chagas disease, Line 3, profile of sample PRMA71, Line 4, Negative control of the reagents used in the PCR reaction (without template DNA), Lines 6 to Line 8, and profiles of samples PRMA85; PRMA92, PRMA71; PRMA123, respectively.
