# Supplementary material for: Oral Chagas disease outbreak by bacaba juice ingestion: A century after Carlos Chagas’ discovery, the disease is still hard to manage
Source: PLoS Negl Trop Dis. 2024 Sep 18;18(9):e0012225. doi: 10.1371/journal.pntd.0012225 (PMC11441692; doi:10.1371/journal.pntd.0012225)
Supplement: S2 Fig — (DOCX) [file pntd.0012225.s002.docx]

**S2 Fig.** Phylogenetic tree of the 33 samples from the outbreak along with 77 samples from another outbreak

**
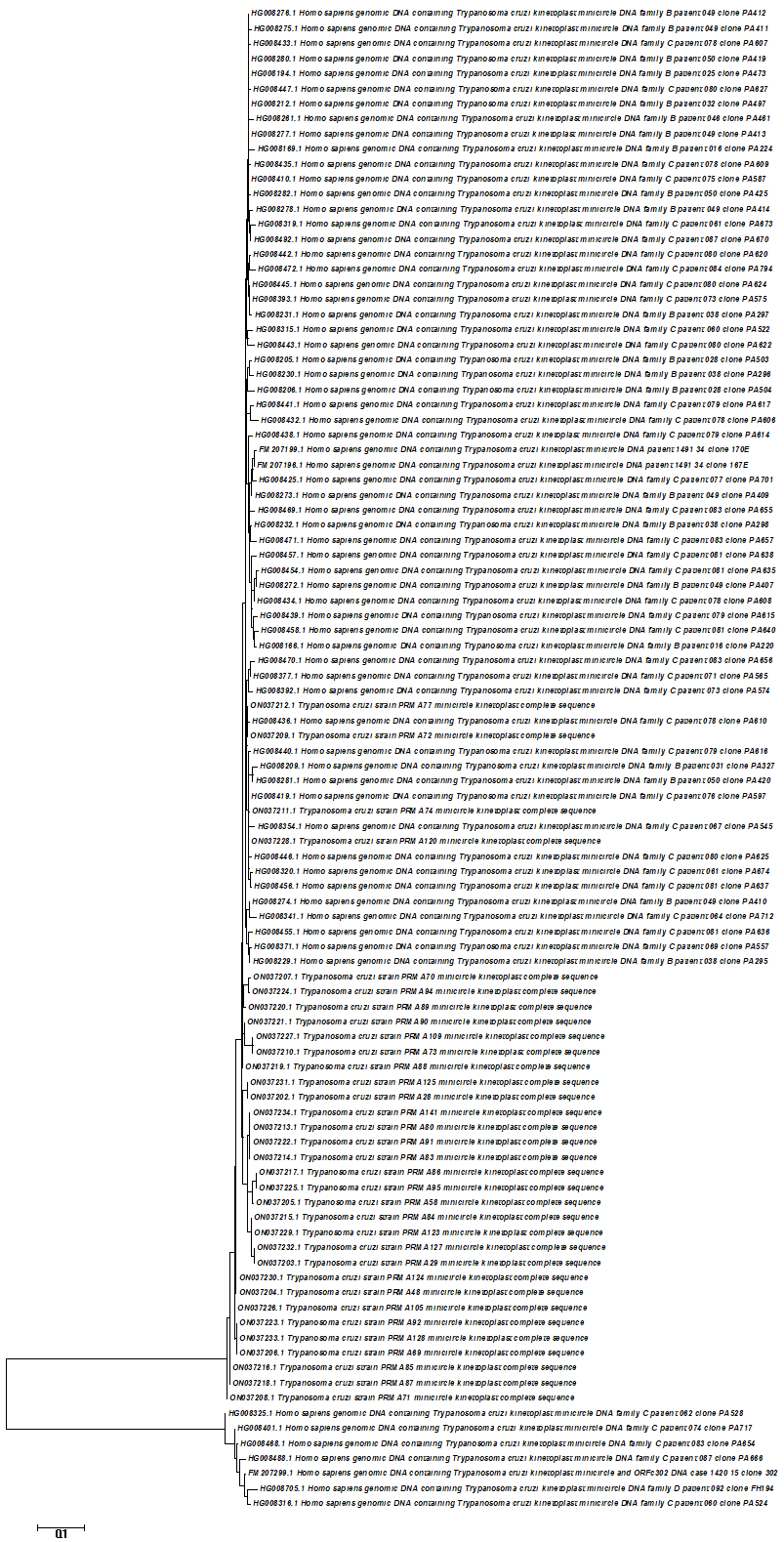
**
