# Supplementary material for: Oral Chagas disease outbreak by bacaba juice ingestion: A century after Carlos Chagas’ discovery, the disease is still hard to manage
Source: PLoS Negl Trop Dis. 2024 Sep 18;18(9):e0012225. doi: 10.1371/journal.pntd.0012225 (PMC11441692; doi:10.1371/journal.pntd.0012225)
Supplement: S1 Text — (DOCX) [file pntd.0012225.s003.docx]

**S1 Text**

**Methodology of Polymerase Chain Reaction**

1. **Extraction of the parasite's genetic material from blood samples and amplification by PCR:** for the DNA extraction process from blood samples, the commercial QIAamp DNA Blood kit (QIAGEN GmbH, Hilden, Germany), were used, according to the manufacturer's instructions. The DNA obtained were quantified in Nanodrop (ND1000 - Thermo Fisher Scientific, Waltham, MA) and stored at -20°C until use. For each PCR reaction, 25 μl of the reaction kit mix containing 0.2 mM of each of the dNTPs, 2 mM MgSO4, 1 × high-fidelity Taq buffer 10X PCR Gold Buffer, 1 U were added to a PCR tube of Platinum Taq DNA polymerase (Life Technologies, Carlsbad CA) and 0.6 μM of each primer specific for the kinetoplast minicircle region (kDNA) of *T. cruzi* and previously described [1], primer Forward Tc121 5'AAATAATGTACGGGKGAGATGCATGA-3' and Reverse Tc122 5'-GGTTCGATTGGGGTTGGTGTAATATA-3', and 1 μL of template DNA. This primer pair amplifies a fragmente of 330 base pairs (bp), which corresponds to the variable region of the kDNA minicircle of *T. cruzi.* The final volume was completed to 50 µL with RNase and DNase free water. The thermal amplification cycle began with 2 min of activation at 95 ºC, followed by 35 cycles of 95 ºC for 40s, 63.5 ºC for 1 minute, 72 ºC for 1 min, and a final extension cycle at 72 ºC for 5 min.
2. **Analysis of PCR amplified products:** to verify the efficiency of the PCR assays and determine the size of the amplified DNA fragment, around 8 μL of the PCR product plus 2 μL of sample buffer (Blue/Orange 6X Loading Dye) was applied to a 2% agarose gel (Promega, USA) of boric acid at 100 V for 1 hour. After electrophoresis, the gels were stained with Gel Red from Biotium (1:1000) for 20 minutes and observed on an ultraviolet transilluminator. Electrophoresis was performed for 1h at 100 Volts in bufer prepared with Tris-Borate (45mM) and 1 mM EDTA (pH 8.0) at a concentration of 0.5X. To determine the size of the PCR products, a 100bp DNA ladder molecular size marker (INVITROGEN, Life Technologies, USA) was included in each run.
3. **Purification of PCR products and sequencing:** purification of the PCR product of the specific 330bp fragment was done using the Wizard SV Gel and PCR Clean-Up System (PROMEGA) kit according to the manufacturer's instructions. To verify purity, the integrity and concentration of the purified product were checked in nanodrop. Sequencing of the purified PCR product was carried out using the dideoxynucleotide chain termination method of Sanger et al [2]. Each DNA strand will be sequenced in both directions of the double strand (positive sense and negative sense) with the “ABI Prism BigDye Kit” on the automatic sequencer ABI 3130 Genétic Analyzer (Applied Biosystems). Samples were sequenced at least twice in each direction of the strand, making a total of four sequences from the same sample. All sequencing were carried out by the company Myleus Biotecnologia Ltda, based in Belo Horizonte-MG.
4. **Computational analysis of sequences:** to check the quality of the sequences, the electropherograms obtained during the sequencing process were analyzed using the ChromasPro data program (Technelysium Pty Ltd). The similarity between the nucleotide sequences obtained was verified by the BLASTn program from the BLAST 2.0 package (Basic Alignment Search Tool) [3], from where The *T. cruzi* reference sequences from Genbank were also selected for comparative analyzes through alignment using the MEGA version 4.0 program [4] and construction of a phylogenetic tree.
5. **Electrophoresis:** was performed for 1 h at 100 Volts in bufer prepared with Tris-Borate (45mM) and 1 mM EDTA (pH 8.0) at a concentration of 0.5X.

**References**

1. Wincker P, Britto C, Pereira JB, Cardoso MA, Oelemann W, Morel CM. Use of a simplified polymerase chain reaction procedure to detect *Trypanosoma cruzi* in blood samples from chronic chagasic patients in a rural endemic area. Am J Trop Med Hyg. 1994 Dec;51(6):771-7. doi: 10.4269/ajtmh.1994.51.771.
2. Sanger F, Nicklen S, Coulson AR. DNA sequencing with chain-terminating inhibitors. Proc Natl Acad Sci U S A. 1977 Dec;74(12):5463-7. doi: 10.1073/pnas.74.12.5463.
3. Altschul SF, Madden TL, Schäffer AA, Zhang J, Zhang Z, Miller W, Lipman DJ. Gapped BLAST and PSI-BLAST: a new generation of protein database search programs. Nucleic Acids Res. 1997 Sep 1;25(17):3389-402. doi: 10.1093/nar/25.17.3389.
4. Tamura K, Dudley J, Nei M, Kumar S. MEGA4: Molecular Evolutionary Genetics Analysis (MEGA) software version 4.0. Mol Biol Evol. 2007 Aug;24(8):1596-9. doi: 10.1093/molbev/msm092. Epub 2007 May 7.
