# Supplementary material for: Oral Chagas disease outbreak by bacaba juice ingestion: A century after Carlos Chagas’ discovery, the disease is still hard to manage
Source: PLoS Negl Trop Dis. 2024 Sep 18;18(9):e0012225. doi: 10.1371/journal.pntd.0012225 (PMC11441692; doi:10.1371/journal.pntd.0012225)
Supplement: S2 Text — (DOCX) [file pntd.0012225.s004.docx]

**S2 Text**

**Methodology of blood smear technique**

1. Add one (01) drop of Giemsa alcoholic solution for each milliliter of buffered water and let it stain for ten (10) minutes.
2. Preparation of buffered water solution: dissolve one (01) gram of phosphate salts in 1000 ml of distilled water.
3. Preparation of phosphated methylene blue aqueous solution: dissolve two (02) grams of phosphated methylene blue in 500 ml of distilled water.
4. Procedures and techniques for pre-staining and staining blood samples (thick drop slide and smear) using the Walker and Giemsa methods:
5. Thick drop, pre-staining procedures: rinse with the aqueous solution of phosphate methylene blue over the thick drops covering all the blood on the slide; then, rinse with the buffered water solution on the slide and let it run gently over the thick drops, removing the excess of the aqueous solution of phosphate methylene blue;
6. Thick drop, staining procedures: place the boron slide on the curved staining plate; gently pour the Giemsa aqueous solution in the proportion of one (01) drop of Giemsa alcoholic solution for each (01) ml of buffered water solution; let it blush for ten (10) minutes, set it strictly on an alarm clock; then gently rinse with the buffered water solution over the slide and let it drain gently over the thick drops, removing all excess Giemsa aqueous solution; place the slides on the wooden board and let them dry at room temperature; then take it to the microscope to read the diagnosis;
7. Blood smear, fixation procedure: drip a few drops of methyl alcohol onto the smear, covering all the blood for 3 to 5 seconds and let it dry;
8. Blood smear, staining procedure: place the borco slide on the curved staining plate; pour the aqueous Giemsa solution in the proportion of one (01) drop of alcoholic Giemsa solution to one (01) ml of buffered water solution; let it blush for 10 to 30 minutes; rinse with buffered water gently over the smear until all excess dye is removed; place the smear (slide) on the wooden board and let it dry at room temperature; then take it to the microscope to read the diagnosis;
