# Supplementary material for: Oral Chagas disease outbreak by bacaba juice ingestion: A century after Carlos Chagas’ discovery, the disease is still hard to manage
Source: PLoS Negl Trop Dis. 2024 Sep 18;18(9):e0012225. doi: 10.1371/journal.pntd.0012225 (PMC11441692; doi:10.1371/journal.pntd.0012225)
Supplement: S3 Text — (DOCX) [file pntd.0012225.s005.docx]

**S3 Text**

**Methodology of safe preparation of bacaba juice**

1. Fruits higienization: harvesting/sifting: make a selection of fruits. Use a collection table or sieve to remove residues (vegetable fragments and stones) and possible insects that may come with the fruit.
2. First wash: the fruits must be washed in running potable water to remove dirt, insects and other residues adhered to the surface of the fruits. It is recommended to wash the fruits in a tank and remove the dirt with a sieve, shaking the fruits well for better washing.
3. Second washing (disinfection): in this stage the fruits are immersed in a chlorinated solution, with a concentration of 150 ppm of active chlorine, for 15 minutes. The sanitizer must be registered with the Ministry of Health and approved for use in contact with food. No bleach or perfumed products should be used. The chlorinated solution can only be used once.
4. Third wash (rinse): must be done in running potable water. This step allows the removal of chlorine residues, which give the product an unpleasant taste and can harm the consumer's health. After cleaning, the fruits should only be stored in sanitized containers covered with transparent plastic, to avoid recontamination by dust, splashes, insects, etc. They must be handled with sanitized hands and utensils and/or equipment.
5. Bleaching: bleaching is a thermal process that promotes a reduction in microbial load, mainly contamination related to *Trypanosoma*. A bath with water at a temperature of 80ºC must be used for 10 seconds.
6. Cooling/softening: at this stage, properly sanitized fruits must be kept in potable water, which may or may not be heated, to cool and soften the pulp, if necessary.
7. Pulping: it is necessary to carry out personal hygiene of the handler and utensils; of the mixer always before starting use and at the end of activities.
8. Packaging: must be performed in clean and intact packaging and these cannot be reused.
9. Storage: they must be packed in plastic bags and kept in a freezer at a temperature between 0 and 4ºC for up to 24 hours. Storing different types of food in the same freezer can be done as long as they are separated, packaged and identified. The freezer must be kept in good working order, clean and well maintained.
10. ALERT: *Trypanosoma* is not destroyed by freezing.
