# Supplementary material for: Oral Chagas disease outbreak by bacaba juice ingestion: A century after Carlos Chagas’ discovery, the disease is still hard to manage
Source: PLoS Negl Trop Dis. 2024 Sep 18;18(9):e0012225. doi: 10.1371/journal.pntd.0012225 (PMC11441692; doi:10.1371/journal.pntd.0012225)
Supplement: S1 Table — (DOCX) [file pntd.0012225.s006.docx]

**S1 Table. Demographic, clinical and laboratory characteristics of 39 cases of acute Chagas disease**

| ID | SEX | AGE (years) | SYMPTOMS | Incubation (days) | Number of days between symptoms and blood smear | Blood smear result | PCR result | IgG ELISA result (1^st^ sample) | IgG IIF result (1^st^ sample) | IgG ELISA result (2^nd^ sample) | IgG HAI result (2^nd^ sample) | IgG ELISA result (4.5 years later) | IgG IIF result (4.5 years later) |
| --- | --- | --- | --- | --- | --- | --- | --- | --- | --- | --- | --- | --- | --- |
| Patients diagnosed through laboratory criteria: positive parasitological findings OR seroconversion of IgG OR 2x increase in titers of IgG IIF | | | | | | | | | | | | | |
| 19 | M | 14 | Fever, lymphadenopathy, asthenia, dyspnea, chest pain, facial edema | 10 | 16 | POS | POS | NEG | INDET 1/40 | POS | INCONC | POS | POS 1/160 |
| 24 | F | 68 | Fever in evening, lymphadenopathy, asthenia, cough, dyspnea, facial edema, diarrhea | 15 | 11 | POS | POS | INDET | INDET 1/40 | POS | POS | - | - |
| 39 | F | 69 | Fever in evening, lymphadenopathy, chill, myalgia, asthenia, abdominal pain. | 14 | 11 | POS | - | NEG | POS 1/160 | POS | POS | POS | INDET 1/40 |
| 1 | F | 10 | Fever in evening, lymphadenopathy, headache, asthenia, facial edema | 17 | 15 | POS | - | POS | POS 1/80 | POS | POS | POS | POS 1/80 |
| 2 | F | 40 | Fever in evening, lymphadenopathy, chill, dyspnea, facial edema, abdominal pain | 14 | 11 | POS | POS | POS | POS 1/160 | POS | POS | POS | INDET 1/40 |
| 3 | M | 58 | Fever in evening, lymphadenopathy, asthenia, cough, dyspnea, diarrhea | 4 | 19 | POS | POS | NEG | NEG | NEG | NEG | NEG | NEG |
| 8 | M | 34 | Fever in evening, lymphadenopathy, chill, myalgia, asthenia | 5 | 27 | POS | POS | POS | POS 1/160 | POS | POS | - | - |
| 10 | F | 39 | Fever in evening, lymphadenopathy, chill, cough, dyspnea, chest pain, lower limb edema, inoculation chagoma?? | 14 | 17 | POS | POS | POS | POS 1/80 | POS | NEG | - | - |
| 11 | F | 15 | Fever in evening, lymphadenopathy, chill, headache, asthenia | 19 | 13 | POS | POS | POS | POS 1/80 | INCONC | INCONC | - | - |
| 12 | F | 40 | Headache, asthenia, cough, dyspnea, facial edema | 19 | 13 | POS | POS | NEG | NEG | NEG | NEG | POS | POS 1/80 |
| 13 | M | 55 | Fever, lymphadenopathy, chill, headache, myalgia, dyspnea, abdominal pain | 2 | 30 | POS | POS | POS | POS 1/80 | POS | POS | - | - |
| 15 | M | 29 | Fever in evening, lymphadenopathy, chill, myalgia, asthenia | 11 | 20 | POS | POS | POS | POS 1/80 | - | - | - | - |
| 16 | F | 52 | Fever in evening, lymphadenopathy, chill, cough, dyspnea, lower limb edema | 12 | 20 | POS | - | INDET | INDET 1/40 | IND. | NEG | POS | POS 1/80 |
| 17 | M | 29 | Fever, lymphadenopathy, chill, asthenia | 18 | 13 | POS | POS | NEG | INDET 1/40 | - | - | - | - |
| 18 | F | 12 | Fever in evening, lymphadenopathy, headache, asthenia, facial edema | 7 | 19 | POS | POS | INDET | POS 1/80 | - | - | - | - |
| 20 | M | 24 | Fever, lymphadenopathy, chill, headache, asthenia, abdominal pain | 15 | 11 | POS | POS | POS | POS 1/80 | POS | POS | - | - |
| 21 | M | 56 | Fever in evening, lymphadenopathy, cough, dyspnea, abdominal pain | 10 | 16 | POS | POS | POS | POS 1/160 | POS | POS (IIF) | IND | NEG |
| 22 | F | 30 | Fever, lymphadenopathy, chill, headache, myalgia, cough, dyspnea, abdominal pain | 13 | 18 | POS | POS | POS | POS 1/80 | POS | POS | POS | POS 1/80 |
| 23 | M | 12 | Fever in evening, lymphadenopathy, headache, myalgia, cough, facial edema | 7 | 19 | POS | POS | POS | INDET 1/40 | POS | POS | POS | POS 1/160 |
| 25 | F | 19 | Fever, lymphadenopathy, myalgia, cough | 15 | 11 | POS | POS | POS | POS 1/80 | POS | POS | NEG | NEG |
| 26 | F | 13 | Fever in evening, lymphadenopathy, headache, myalgia, facial edema, abdominal pain | 10 | 16 | POS | POS | NEG | NEG | INCONC | INCONC | POS | POS 1/80 |
| 27 | F | 32 | headache, cough, dyspnea, facial edema, abdominal pain | 17 | 15 | POS | POS | POS | POS 1/80 | POS | POS | POS | POS 1/80 |
| 32 | F | 1 | Fever, lymphadenopathy, facial edema, abdominal pain, diarrhea | 15 | 23 | POS | POS | NEG | INDET 1/40 | NEG | NEG | POS | POS 1/160 |
| 5 | M | 33 | Fever in evening, lymphadenopathy, chill, myalgia, asthenia | 16 | - | - | POS | POS | INDET 1/40 | POS | POS IIF 1/160 | POS | POS 1/160 |
| 33 | M | 14 | Fever, lymphadenopathy, | 28 | - | - | POS | NEG | POS 1/80 | POS | POS | - | - |
| 37 | F | 68 | Chill at evening, asthenia, dyspnea, lower limb edema, facial edema | 12 | - | - | - | INDET | INDET 1/40 | POS | POS | POS | POS 1/160 |
| Patients diagnosed through epidemiological criteria: clinical findings AND epidemiological link AND at least one positive IgG finding | | | | | | | | | | | | | |
| 4 | M | 28 | Fever at evening, lymphadenopathy, chill, myalgia | 5 | 26 | NEG | - | POS | POS 1/80 | POS | POS | NEG | NEG |
| 6 | M | 37 | Fever at evening, lymphadenopathy, chills, myalgia, asthenia | 24 | 8 | NEG | - | POS | POS 1/80 | POS | POS | POS | POS 1/80 |
| 7 | M | 50 | Fever, lymphadenopathy, asthenia | 26 | 7 | NEG | POS | POS | POS 1/80 | POS | POS | NEG | NEG |
| 9 | M | 28 | Fever in evening, lymphadenopathy, headache, myalgia, asthenia, dyspnea | 15 | 17 | NEG | POS | POS | POS 1/160 | POS | POS | POS | POS 1/80 |
| 14 | M | 4 | Fever in evening, lymphadenopathy, chill, facial edema | 13 | 13 | NEG | POS | POS | INDET 1/40 | POS | POS | POS | POS 1/80 |
| 28 | M | 48 | Fever in evening, lymphadenopathy, chill, myalgia, asthenia | 12 | 11 | NEG | POS | POS | POS 1/160 | POS | POS | POS | POS 1/80 |
| 29 | M | 23 | Fever at evening, lymphadenopathy, chill, myalgia, asthenia | 30 | - | - | POS | POS | POS 1/160 | POS | POS | - | - |
| 34 | F | 35 | Fever in evening, lymphadenopathy, chill, headache, myalgia, dyspnea, chest pain, lower limb edema, facial edema | 16 | 7 | NEG | POS | INDET | POS 1/160 | INCONC | INCONC | NEG | NEG |
| 38 | F | 41 | Fever in evening, lymphadenopathy, myalgia, asthenia, edema MII, facial edema | 7 | - | - | - | POS | POS 1/80 | - | - | - | - |
| Patients without diagnostic criteria: clinical findings AND epidemiological link AND negative tests | | | | | | | | | | | | | |
| 30 | M | 0.8 | Fever | 16 | 22 | NEG | POS | NEG | NEG | NEG | NEG | NEG | NEG |
| 31 | F | 11 | Fever, lymphadenopathy, asthenia | 13 | - | - | POS | NEG | NEG | - | - | NEG | NEG |
| 35 | F | 78 | Fever in evening, lymphadenopathy, chill, myalgia, asthenia, cough, dyspnea, facial edema, abdominal pain | 11 | 22 | NEG | POS | NEG | NEG | NEG | NEG | - | - |
| 36 | F | 63 | Fever in evening, lymphadenopathy, asthenia, cough | 15 | 16 | NEG | POS | NEG | NEG | NEG | NEG | NEG | NEG |
| ID, patient identity number; M, male; F, female; PCR, polymerase chain reaction; ELISA, enzyme-linked immunosorbent assay; HAI, hemagglutination inhibition assay; IIF, indirect immunofluorescence test; POS, positive; NEG, negative; INDET, indeterminate; INCONC, inconclusive. | | | | | | | | | | | | | |
