# Supplementary material for: Oral Chagas disease outbreak by bacaba juice ingestion: A century after Carlos Chagas’ discovery, the disease is still hard to manage
Source: PLoS Negl Trop Dis. 2024 Sep 18;18(9):e0012225. doi: 10.1371/journal.pntd.0012225 (PMC11441692; doi:10.1371/journal.pntd.0012225)
Supplement: Attachment — Submitted filename: Response_to_reviewers.doc [file pntd.0012225.s009.doc]

**LETTER WITH REVISIONS**

**MANUSCRIPT NUMBER PNTD-D-24-00693**

July 18, 2024.

*Andrés F. Henao-Martínez, M.D.*
Academic Editor

*Susan Madison-Antenucci*
Section Editor

Dear Editors,

We want to thank you so much for your time, support and all helpful suggestions regarding our paper entitled “**An outbreak of Chagas disease through oral transmission: 115 years after discovery by Carlos Chagas, the disease is still hard to manage**." Your comments and suggestions helped us build a better paper. In this revised version of our manuscript, we incorporated all reviewer´s suggestions, including some adjustments in references, a new table with the resume of results, an image, and a flowchart on the methodology. Additionally, we revised the manuscript to provide greater clarity of information. Below is the list of answers to each reviewer’s comments:

| **METHODS** | | |
| --- | --- | --- |
| **REVIEWER** | **COMMENTS** | **RESPONSE** |
| Reviewer #1: | The methods used to develop the study were adequate. | Thank you for your comment. |
| Reviewer #2: | PITs are a strategy used by the Oswaldo Cruz Foundation (FIOCRUZ) to monitor vectors. PITs are epidemiological surveillance strategies. And not from FIOCRUZ. | Accepted. Change in line 208-209: “PITs are epidemiological surveillance strategies.” |
| Reviewer #3: | -Are the objectives of the study clearly articulated with a clear testable hypothesis stated? YES -Is the study design appropriate to address the stated objectives? YES -Is the population clearly described and appropriate for the hypothesis being tested? YES -Is the sample size sufficient to ensure adequate power to address the hypothesis being tested? YES -Were correct statistical analysis used to support conclusions? YES -Are there concerns about ethical or regulatory requirements being met? NO | Thank you for your comment. |
| Reviewer #4: | Overall, the study's objectives are clearly articulated, providing a well-defined and testable hypothesis. However, it would be appropriate for the authors to explain why they considered analyzing only two time points: before the treatment and 4.5 years after the treatment. Why did they not consider other time points after the treatment? (lines 167-168). | Accepted. Change in lines 178-180: “It was not possible other time point analysis due to municipality`s resource limitations.” |
|  | In general, the study population is briefly described, but in another section of the manuscript. The authors should conduct an extensive review of the text, as information that should be in the methodology section is found in other sections. For example, the data contained in lines 221-222 (related to the results section) should be in the methodology. It would also be useful to include a table in the methodology section that gathers data on the number of patients, their age, gender, the time taken to receive treatment, and the therapeutic regimen they underwent. This information is present in the text but in a different section and not very detailed, which makes the presentation of the data quite confusing.  It is important for the methodology to be more thoroughly explored. Information about the outbreak is neither in the introduction nor the methodology but is presented throughout the results. The authors should review the manuscript text, which needs to be restructured. Additionally, we need more data about the outbreak: where does it occur? Provide a map. How many people are involved? Provide the latitude and longitude of the location. Describe the characteristics of the location, etc. Some of this information is in the results section and should be in the methodology, while other information is missing from the manuscript and should be included. In line 174, the authors mention criteria established in the literature and guidelines but do not specify which ones. Please cite them. Suggestion: present the criteria (lines 174-178) in a table. The authors describe the cPCR in the Supporting Information section. | Accepted. Change in lines 171-175: We insert the location information’s, with geographic location. Change in lines181-186: we insert inclusion and exclusion criteria. Change in line 192: We provide the missing reference. Change in lines 223-225: We create an flowchart with the resume of the methodology (Fig1). Change in lines 316-318: We created a table summarizing the findings (Table 2) and put the big table in supporting information (Table 3).  The map of the location already exists (Fig3). |
| Reviewer #5: | The objectives are clear and practical, the study population is delimited and the size of the population does not affect the results. Ethics protocols could be better clarified, such as the use or not of the 'Plataforma Brasil'. In 'supporting information' all protocols need details and not just the PCR. | Accepted. Change in lines 169-170: “through submission through the ‘Plataforma Brasil’”. Change in lines 957-1028: We add the blood smear technique, triatomine analysis, and the safe production of juice protocol in Supporting information. |
| Reviewer #6: | yes to all the above.  No concerns about ethics. | Thank you for your comment. |
| **RESULTS** | | |
| Reviewer #1: | The resulsts are well presented. | Thank you for your comment. |
| Reviewer #2: | After an interval of 4.5 years, ELISA and IIF IgG were performed on 26 individuals, which showed that serological cure had been achieved (negative tests) in 34.6% of the cases (9/26), while decreased IgG titers were found in 15.3% of the cases (4/26). What about the others evaluated? | Accepted. Change in lines 350-352: “For 26.9% of cases (7/26) there was an increase in IgG titers and 15.3% of cases (4/26) maintained the same level. In 7.6% of cases (2/26) there were seroconversion.” |
|  | After this outbreak, a series of training sessions were conducted among the municipality's (...) I suggest this part is in the results section | Accepted. Change in lines 354-360. |
| Reviewer #3: | -Does the analysis presented match the analysis plan? YES -Are the results clearly and completely presented? YES -Are the figures (Tables, Images) of sufficient quality for clarity? YES | Thank you for your comment. |
| Reviewer #4: | Since there are missing details in the methodology, the results are consequently presented in a confusing manner. For example, subsection 3.1 (lines 204-222) should be rewritten in the methodology. It is also important to include how the 85 individuals investigated due to the outbreak were assessed (line 221). | Accepted. Changes already cited in comments above. |
|  | Additionally, subsection 3.2 (lines 224-267) should also be in the methodology, and more information should be added, such as: what is the climate type of the location? What type of vegetation is there? | Accepted. Changes already cited in comments above. |
|  | Line 235: The authors should add an image of the 'mucura.' | Accepted. Change in lines 275-278. Added the image (Fig5). |
|  | Lines 237-238: What are the predominant species of triatomines in the study area? | Accepted. Change in lines 267-270: The most frequently species in the state are from genera *Pangystrogylus* and *Rhodnius* [24]. However, the data is old, as routine monitoring is not being performed by the municipalities and the state. |
|  | Line 247: What is the seasonal period of bacaba? This should be mentioned in the text. | Accepted. Change in line 284-285: “which occurs between December and April” |
|  | Lines 259-260: How was the infection analysis of the triatomines conducted? | Accepted. We include the protocol Supporting information. |
|  | Lines 268-275: Suggestion: a graph could be plotted showing the frequencies of the symptoms. | Accepted. Change in lines 316-318: We created a table summarizing the findings (Table 2) and put the big table in supporting information (Table 3). |
|  | Table 2 (line 277) is too extensive, making it difficult to understand the results and compare the different diagnoses. Suggestion: the table could be split to separately present the different criteria and the results before treatment and 4.5 years after treatment. | Accepted. We put the big table in supporting information (TABLE 3). |
|  | The first information about the sequencing was only presented in the results (line 289). There should be information about the sequencing in the methodology. Why and how was the sequencing performed? Were they able to identify the different DTUs of T. cruzi? | This methodology of PCR and sequency is presented in supporting information. We did not perform additional experiments to determine DTUs, since the primer pairs we used are used to diagnose the presence of the parasite in both the acute and chronic phases of Chagas disease. The sequencing performed was to confirm the results obtained in the PCR. To determine DTUs, we would need to use specific markers for genotyping. |
| Reviewer #5: | The results section is complete and adequately presents the same | Thank you for your comment. |
| Reviewer #6: | Figure 1 could be specifically cropped to show the trypomatigote more clearly. | Accepted. Change in lines 239-242: We choose another photo from the exam (Fig 2). |
| **CONCLUSION** | | |
| Reviewer #1 | The work is relevant and the conclusions were supported by the data presented. Limitations of the analysis were not described. | Accepted. Change in lines 578-581. “and the neglect of governments to perform the basic testing and adequate follow up of affected people. The latter ended up being the limiting factor of this study, as it was not possible to perform basic cardiological assessment tests in all patients, serial serologies in shorter times and other molecular biology samples” |
| Reviewer #2: | (No Response) | --- |
| Reviewer #3: | -Are the conclusions supported by the data presented? YES -Are the limitations of analysis clearly described? YES -Do the authors discuss how these data can be helpful to advance our understanding of the topic under study? YES -Is public health relevance addressed? YES | Thank you for your comment. |
| Reviewer #4: | In the conclusion of the manuscript, the authors call for a reflection on Chagas disease, which is highly important, especially with the increase in cases and outbreaks observed in the region and in other endemic and non-endemic areas. However, the authors should discuss more about the methodologies proposed for the molecular diagnosis of Chagas disease, considering they propose the methodology used in the study, which is conventional PCR (described in 1989 by Wincker), as the gold standard method. Although this methodology is well established, the authors do not explore it much throughout the manuscript, nor do they discuss and compare it with other already described molecular methodologies. Reading the 2nd Brazilian Consensus on Chagas Disease, 2015, published in 2016, as well as other related articles, could enrich the discussion about the proposal made by the authors. I also recommend the reference Moreira et al., 2023 (Validation of the NAT Chagas IVD Kit for the Detection and Quantification of Trypanosoma cruzi in Blood Samples of Patients with Chagas Disease), as it deals with a commercial kit approved by ANVISA for molecular diagnosis through real-time PCR of patients with Chagas disease. | Accepted. We proposed that any PCR method be standardized for routine use. In lines 473-474 we cite a systematic review and meta-analysis published in 2023 that failed to demonstrate which method was more effective due to the heterogeneity of the studies. Regarding the commercial kit, we thank you for the reference and included it in the discussion. Change in lines 467-470: “as well as the scarcity of commercial kits for use in routine surveillance. To date, only one kit has been approved for use in Brazil, the NAT Chagas Kit®, developed by the Oswaldo Cruz Foundation (FIOCRUZ) in partnership with the Institute of Molecular Biology of Paraná (IBMP), and its use is still far from being universal [57] |
|  | The arguments in lines 380-384 and 389-392 are repetitive and redundant. The authors should combine them into a single justification for not performing IgM analysis in these patients. | Accepted. Change in lines 442: “due to the limitations mentioned above” |
|  | Lines 407-408: Could it not be related to therapeutic failure? The MULTIBENZ study (Bosch-Nicolau et al., 2024) explores this | Accepted. Change in lines 458-461: “This finding brings to light the possibility of hypotheses such as pre-exposure (as some of the patients already had positive IgG in the first sample), or re-exposure to the pathogen. This could mean that patients already had the indeterminate chronic form of the disease, contributing to therapeutic failure. |
|  | Lines 412-414: Currently, the commercial NAT Chagas kit (Moreira et al., 2023) is in circulation. | Accepted. Change in lines 467-470: mentioned above. |
|  | Lines 435-438: What are the most common DTUs? | this information is presented in lines 485-488 |
| Reviewer #5: | I found the discussion extensive and could present a clear and objective discussion. | Thank you for your comment. We chose to submit this work in this journal due to its non-limitation. The topic is very extensive, and we believe we should explore it. |
| Reviewer #6: | Conclusions are supported by the data. Limitations of diagnostic tests are discussed. Public health relevance cleearly highlighted. | Thanks for your comment. |
| **EDITORIAL AND DATA PRESENTATION MODIFICATIONS** | | |
| Reviewer #1: | The study presents health public relevance. Some minor modifications are required: 1. Present a paragraphy describing the limitations of the study | Accepted. Change in lines 552-556: “The limitations of this study are all related to the resource limitations of the municipalities and the state. It was not possible to perform an adequate cardiological assessment on all patients, restricting the clinical analysis; it was not possible to carry out other collections in a shorter period of time, making it impossible to analyze the serological curve; nor was it possible to collect new PCR samples.” |
|  | 2. The discussion is too long and repetitive. It could be reduce to 2/3 lenght | Thank you for your comment. We chose to submit this work in this journal due to its non-limitation. The topic is very extensive, and we believe we should explore it. |
|  | 3. Add a suggestion of protocol containing PCR as a diagnosis test to ACD | We propose in the conclusion that this method should be performed in all cases with negative parasitology. |
| Reviewer #2: | (No Response) | --- |
| Reviewer #3: | (No Response) | --- |
| Reviewer #4: Minor modifications: | Abstract: Line 46 – Correct “Ita” to “Its” | Accepted. Changed. |
|  | Introduction: Lines 101-103 - Lack of reference for the information provide herein. Please include. | Accepted. Change in line 104: inserted a reference [1,3]. |
|  | Lines 137-139 - Why not use qPCR methodology instead of cPCR? | cPCR was the only one available at the time. |
|  | Line 144: It would be interesting to highlight the challenges of treatment with these medications in chronic patients. | Thank you for your comment. We kindly disagree with your suggestion, as we understand that the work is focused on the acute form of the disease. |
|  | Discussion: Lines 320-322 - Lack of reference for the information provide herein. Please include. | Accepted. Changed. Inserted a reference. |
|  | Line 378: Lack of reference for the specificity of serological tests. Please include. | Accepted. Adjust the reference. |
|  | Supplementary: Line 803: What is the size in base pairs of this product? | Accepted. We include this information supporting information (lines 902-903). The size of the amplified fragment is 330 base pairs (bp), which corresponds to the variable region of the kDNA minicircle. |
|  | Line 812: Is it just boric acid or is it a buffer with Tris, boric acid, and EDTA? Also, what concentration is used? | Please consider that electrophoresis was performed for 1 h at 100 Volts in bufer prepared with Tris-Borate (45mM) and 1 mM EDTA (pH 8.0) at a concentration of 0.5X. We include this information supporting information (lines 914-916) |
|  | About the results of cPCR: Is there a representative gel for this supplementary information? | We have added Figure 5 containing the PCR amplification result of five randomly chosen samples for illustrate the results obtained for the other samples. In some samples, we observed the presence of nonspecific bands, despite having followed the protocol described by the author (lines 919-926) |
|  | Were negative controls for extraction and PCR used, as they are crucial for molecular diagnosis? | We use positive and negative controls as the standard norm in our laboratory for all our experiments, including the process of extracting genetic material from the clinical sample to PCR amplification and electrophoresis test. |
|  | After sequencing, which DTUs were found in these patients? | We did not perform additional experiments to determine DTUs, since the primer pairs we used are used to diagnose the presence of the parasite in both the acute and chronic phases of Chagas disease. The sequencing performed was to confirm the results obtained in the PCR. To determine DTUs, we would need to use specific markers for genotyping. |
| Reviewer #5: | Minor revision. |  |
| Reviewer #6: | A few minor changes (mainly clarification) are recommended before acceptance 1.Line 85. “executability” - is practicality a better term? | Accepted. Change in line 84: “feasibility”. |
|  | 2.Line 108. Please clarify what is meant by “although a representative magnitude for society remained”. | Accepted. Change in lines 107-109: “as Brazil continues to be the country with the highest estimated number of cases, and number of deaths from the disease” |
|  | 3.Line 112.  Please specify which forms of transmission remain a challenge in the authors’ opinion: oral, transplacental, sexual, via blood transfusion or via organ transplantation? | Accepted. Change in lines 113-114: “especially vertical, oral and post-transplant” |
|  | 4.Lines 205 to 212. As written, it is not clear if the third malaria blood smear showed the presence of trypomastigotes and that this observation prompted a re-examination of the first two smears, which were subsequently also found to be positive for Trypanosoma cruzi. | Accepted. Change in lines 236-237: “there was no need to perform the third sample” |
|  | 5.Line 217.  Would it be possible to enlarge and crop the area of the blood smear to show the trypomastigote while retaining a reasonable resolution in Figure 1? | Accepted. Change in lines 239-242: We modified the figure and crop the parasite (Fig2). |
|  | 6.Line 224. Not all readers will know that a quilombo is a settlement originally founded by fugitive slaves of African origin. Please expand to clarify. | Accepted. Change in line 251: “which is a type of community originally founded by fugitive slaves of African origin.” |
|  | 7.Line 274. I presume the facial edema was not associated with Romaña’s sign? It might be useful to specifically state that it was absent (if that was the case) as this reduces the possibility of vector transmission in these cases. | Accepted. Change in line 315: “There were no patients with Romaña’s sign” |
|  | 8.Line 291-292 and supporting information. Comment on how cross-contamination was ruled out as a cause of a positive PCR result when all other tests were negative. | We would like to clarify that during all PCR tests we take all possible precautions to avoid cross-contamination. We use a 10% hypochlorite solution to clean equipment, pipettes and benches. In addition, our laboratory has separate rooms for extraction DNA; room equipped with ultraviolet light for irradiation of pipettes and plastic consumables exclusive for PCR; room for preparation of reagents; room for addition of template DNA; room where the thermocyclers are located and exclusive room for preparation of gels and electrophoresis. Additionally, we used a negative control tube in each experiment containing all the reagents used in the amplification reaction, completing the reaction volume with sterile Mili Q water instead of the template DNA. We also used a positive control tube, that is, with a template DNA originating from samples known to be positive for the organism being investigated. |
|  | 9.Lines 489 to 490.  Some specific details of the recommended pasteurisation method (and references to experimental validation) might be helpful here, since this does not specifically clarify how this differs from the method on line 255 where the liquid is slightly warmed but not boiled | Accepted. Change in lines… of Supporting information: Fruit processing is included in the safe practice protocol produced by SEBRAE and was included in the references [26]. |
|  | 10. References. The list is comprehensive and appropriate.  Please ensure that all titles are in sentence case not title case and that all species names are italicised | Accepted. We made an review of all these questions and changed. |
| **SUMMARY AND GENERAL COMMENTS** | | |
| Reviewer #1: | (No Response) | --- |
| Reviewer #2: | The article is well written and with all the relevant information. However, I believe that some aspects could be improved.  Abstract: -Ita expansion to Other Please check the text. I believe there is something written wrong. | Accepted. Changed to its. |
| Reviewer #3: | Dear authors,  The manuscript is well structured, with appropriate language and references. The outbreak of oral infection is well documented, followed over a long period.  As strengths I highlight: - the importance of PCR for diagnosing acute cases of Chagas disease; - the importance of PITs for contributing to the sampling of triatomine species; - long period of patient follow-up.  As weakness I highlight: the lack of a PCR test at the end of the 4.5 years of follow-up. I believe it would be interesting to observe the efficiency of PCR at a later stage of infection.  Below are some small suggestions to contribute to the work: | Accepted. Change in lines: 552-556: we insert an text about weakness. |
| Reviewer #3: | Line 45: I believe that "however" is not the best conjunction considering the first sentence. As a suggestion, I believe it could be kept in the same sentence: “(...) subtropical zones, and even more (...)” | Accepted. Changed |
|  | Line 46: Change “Ita” to “It’s” | Accepted. Change to its. |
|  | Line 92: Change “Trypanosoma cruzi” to “Trypanosoma cruzi (Chagas, 1909) (Kinetoplastida, Trypanosomatidae)” | Accepted. Changed. |
|  | Line 115: Change: “Panstrongylus geniculatus” to “Panstrongylus geniculatus (Latreille, 1811) (Hemiptera, Triatominae)”. Please add the author and year to other scientific names that are mentioned for the first time. | Accepted. Changed. |
|  | Line 317: Please show the currency of the value | Accepted. Changed. |
|  | Line 427: At the beginning of the sentence, the species name is not abbreviated | Accepted. Changed. |
| Reviewer #4: | This paper provides important information and data about Chagas disease and the most relevant transmission methods at the moment. However, the authors should conduct an extensive review of the manuscript as it needs restructuring. Additionally, enriching the discussion with more current data is important so they can propose cPCR methodology as the gold standard. | Accepted. We have performed an extensive review and hope that after this changes the article will be ready for acceptance. As we mentioned previously, we did not propose the conventional method as the gold standard. We proposed that any PCR method be standardized for routine use. |
| Reviewer #5: | I understand the problem and see that the manuscript is necessary. | Thank you for your comment. |
| Reviewer #6: | This is a well presented and clearly written article describing the diagnosis, treatment and control of an outbreak of Chagas disease via oral transmission in northeastern Brazil.  The contents of this article will be of considerable interest to the readership, particularly the extensive discussion of the limitations of currently used diagnostic techniques and test of cure through parasitological, serological and molecular genetic methods.  The need for better, safer drug treatments and the need for implementation of effective food safety measures, appropriate for areas of extreme poverty are also appropriately highlighted. | Thank you for your comment. |

Thank you for accepting this manuscript.

Sincerely,

Eudes Alves Simões Netoà Noite e
Federal University of Maranhão
Estrada Pinheiro/Pacas, Km 10, S/N, Enseada.

Pinheiro, Maranhão, Brazil. 65.200-000.
